# Supplementary material for: Lignin-polysaccharide interactions in plant secondary cell walls revealed by solid-state NMR
Source: Nat Commun. 2019 Jan 21;10:347. doi: 10.1038/s41467-018-08252-0 (PMC6341099; doi:10.1038/s41467-018-08252-0)
Supplement: Supplementary file 1 — Supplementary Information [file 41467_2018_8252_MOESM1_ESM.pdf]

Supplementary Information

Lignin-Polysaccharide Interactions in Plant Secondary Cell Walls Revealed by Solid-State NMR

Kang et al.

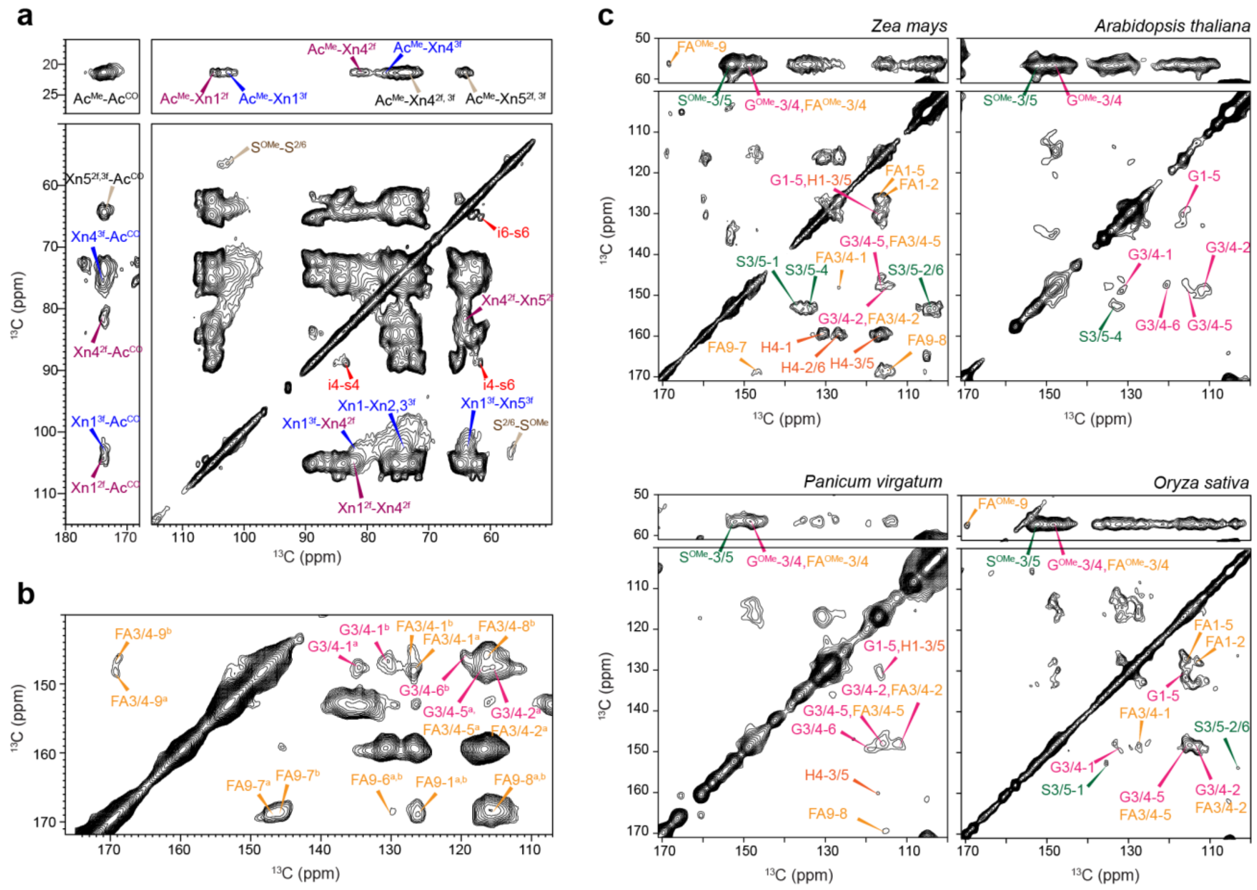

Supplementary Figure 1. Structural and compositional heterogeneity of secondary cell walls. a, Two-fold and three-fold xylan have distinct chemical shifts as shown in 100 ms DARR spectra of *Zea mays*. The well-resolved signals of 2-fold and 3-fold xylans are labeled in purple and blue, respectively. **b**, Structural polymorphism of lignin residues. Two subtypes have been resolved for both G and FA as annotated using superscript a and b. **c**, The lignin regions of 2D DARR spectra of the secondary cell walls of four different plants. Compared with the *Zea mays* (maize), *Arabidopsis thaliana* does not have FA, the *Panicum virgatum* (switchgrass) has reduced amount of S and the *Oryza sativa* (rice) has negligible H signals.

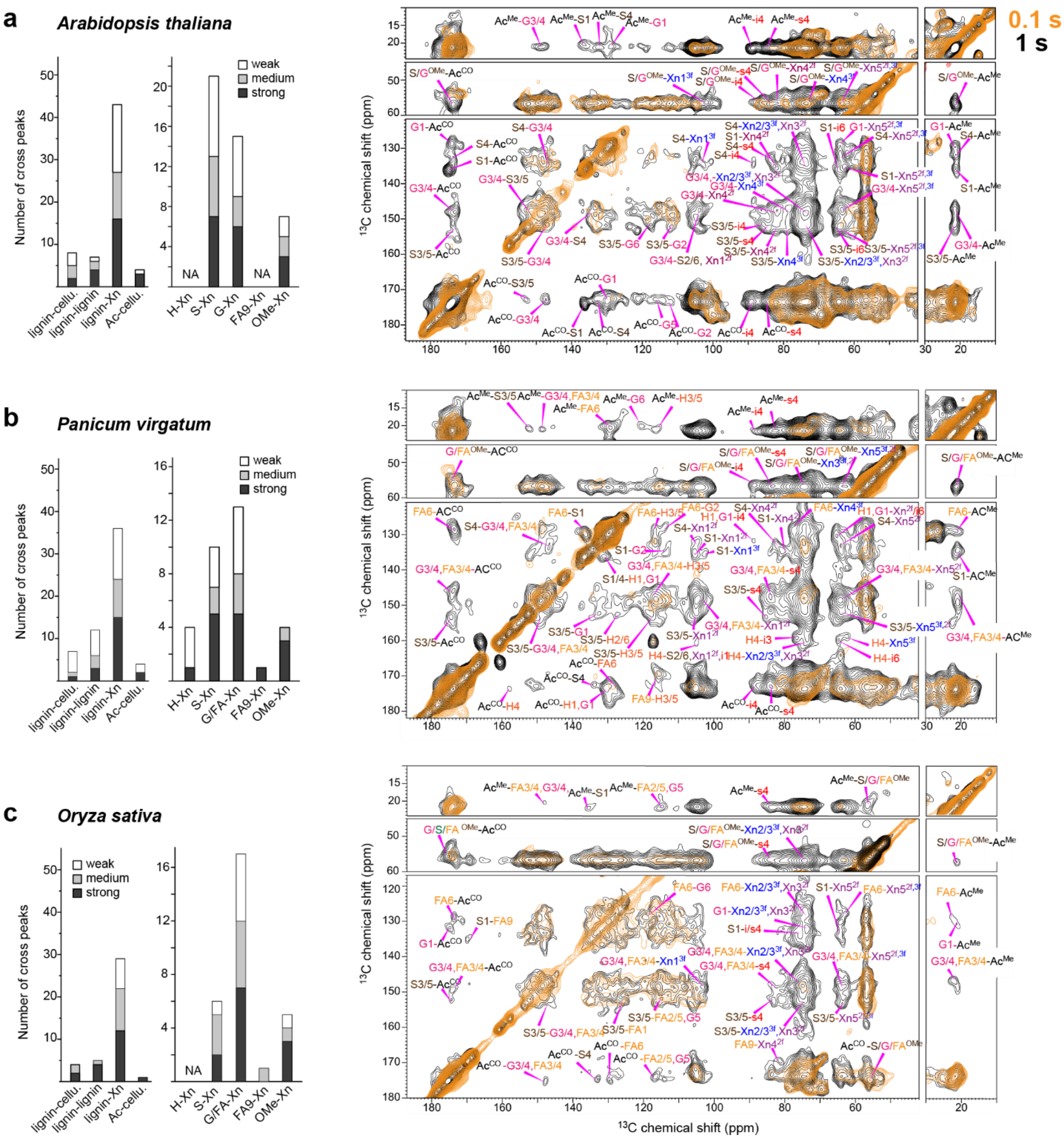

Supplementary Figure 2. Intermolecular interactions of polymers in intact plant stems. The summary bar diagrams and the gated 2D ^{13}C-^{13}C correlation spectra are shown for **a**, *Arabidopsis thaliana*, **b**, *Panicum virgatum* (switchgrass), and **c**, *Oryza sativa* (rice). 100 ms spectra (yellow) mainly detect the intramolecular correlations, and the 1 s (black) spectra show many intermolecular cross peaks. Note that the FA/G is only G in *Arabidopsis*. Only the intermolecular cross peaks are labeled. Source data are provided as a Source Data file.

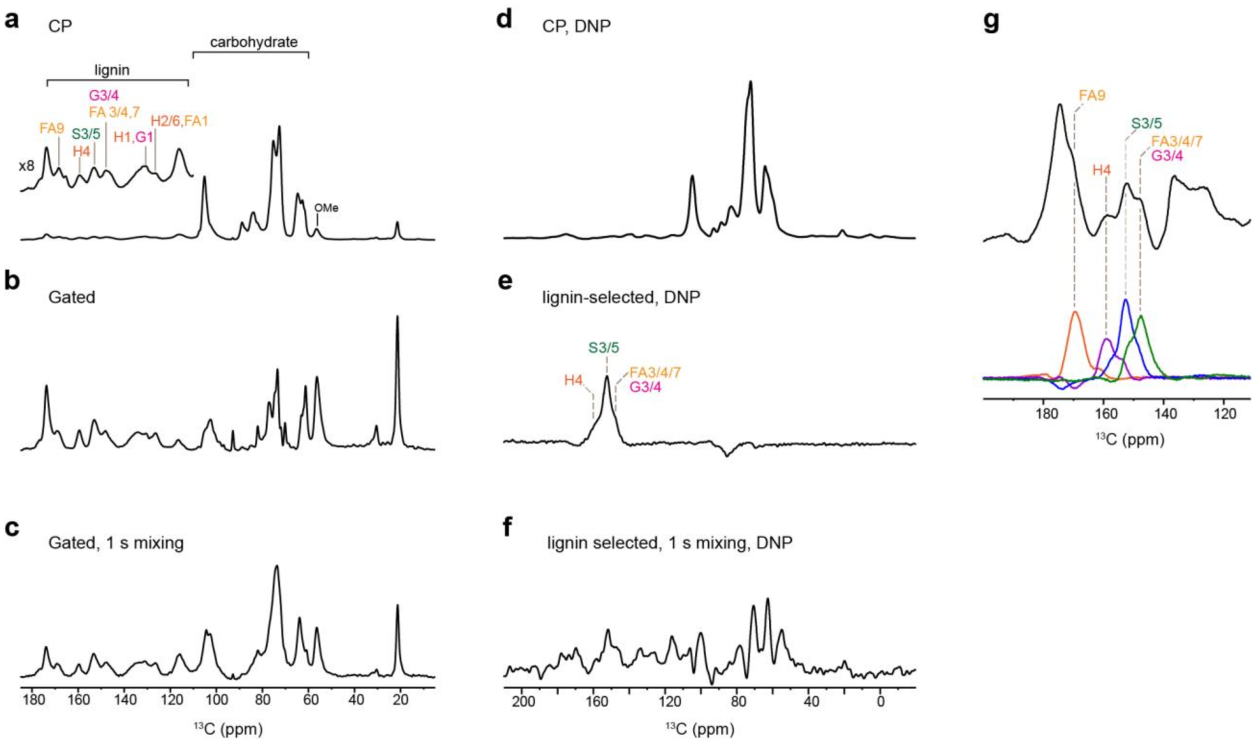

Supplementary Figure 3. Selection of lignin signals using dipolar and frequency filters. a, Representative 1D ^{13}C CP spectrum shows predominant signals of carbohydrates. **b**, The gated spectrum enhances the relative intensity of lignins. **c**. Gated spectrum with 1 s PDSD mixing time to transfer polarization from lignins to carbohydrates. **d**, DNP-enhanced ^{13}C CP spectrum. **e**, DNP-assisted lignin edited spectrum using dipolar and frequency filters cleanly select the lignin signals against the dominant carbohydrate peaks. **f**, DNP-assisted lignin selected spectra with 1 s mixing time. The selected signals have contributions from all the four major lignin units and is used for measuring lignin-edited 2D ^{13}C-^{13}C correlation spectra. **g**, DNP-assisted single-site selection using the dipolar and frequency filters. The DNP spectra were measured on a 600 MHz/395 GHz spectrometer under 10 kHz MAS.

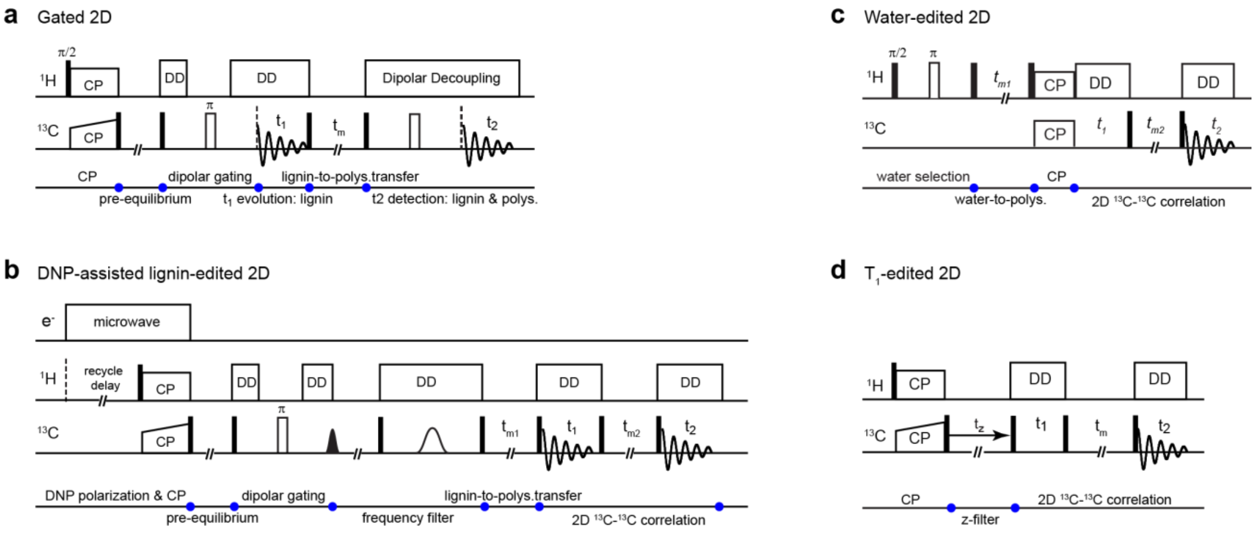

Supplementary Figure 4. NMR pulse sequences for determining lignin-carbohydrate interactions. a, Gated 2D ^{13}C-^{13}C correlation experiment for detecting lignin-carbohydrate correlations, **b**, DNP-assisted lignin-edited 2D ^{13}C-^{13}C correlation experiment that shows the signals of lignin-proximal carbohydrates. A shutter is used to regulate the duration of microwave. **c**, Water-edited 2D ^{13}C-^{13}C correlation experiment, **d**, 2D ^{13}C-^{13}C correlation experiment with z-filter time for measuring ^{13}C-T_1.

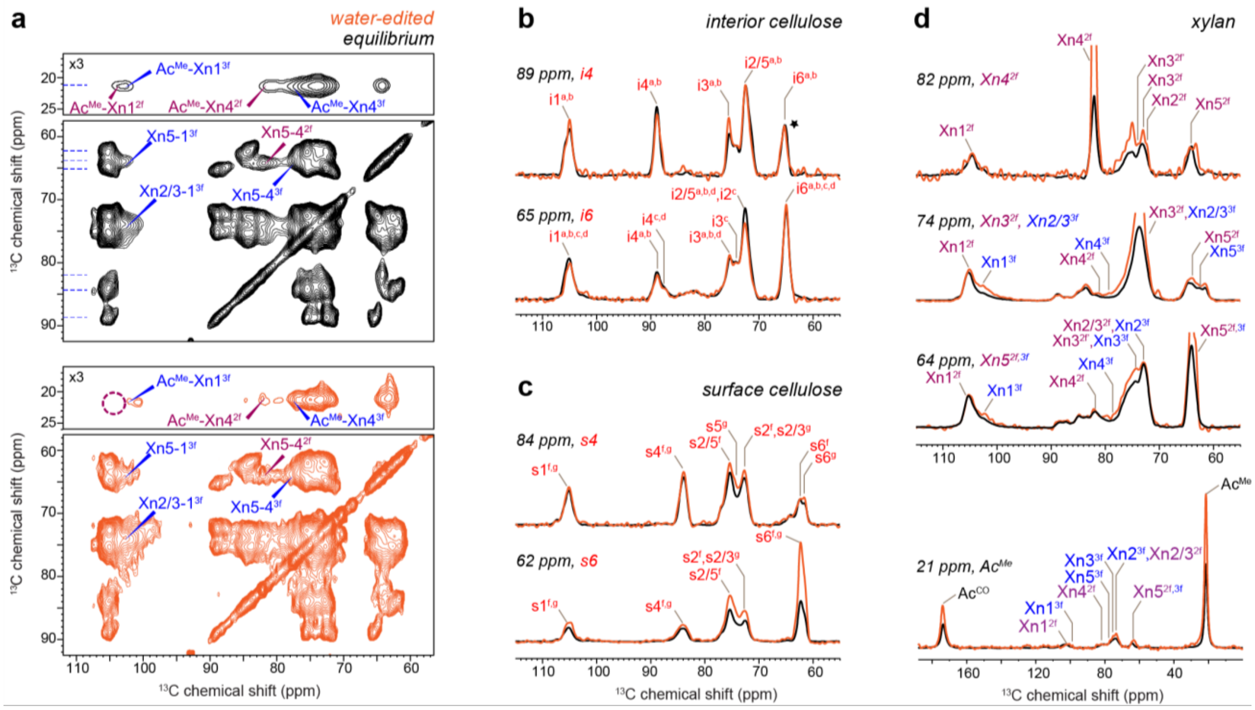

Supplementary Figure 5. Water-edited 2D ^{13}C-^{13}C correlation spectra of maize. a, Comparison of 2D water-edited (orange) and control (black) ^{13}C-^{13}C correlation spectra. The plotting base level is 3-times higher for polysaccharides than the acetyl region. The blue dash lines indicate the positions at which the ^{13}C cross sections are extracted and compared. The Representative 1D ^{13}C cross sections are categorized as **b**, interior cellulose, **c**, surface cellulose and **d**, xylan. All the cross sections are normalized by the i4-6 peak (asterisk). The 3-fold xylan has enhanced intensity in the water-edited spectra, indicating better interactions with water molecules.

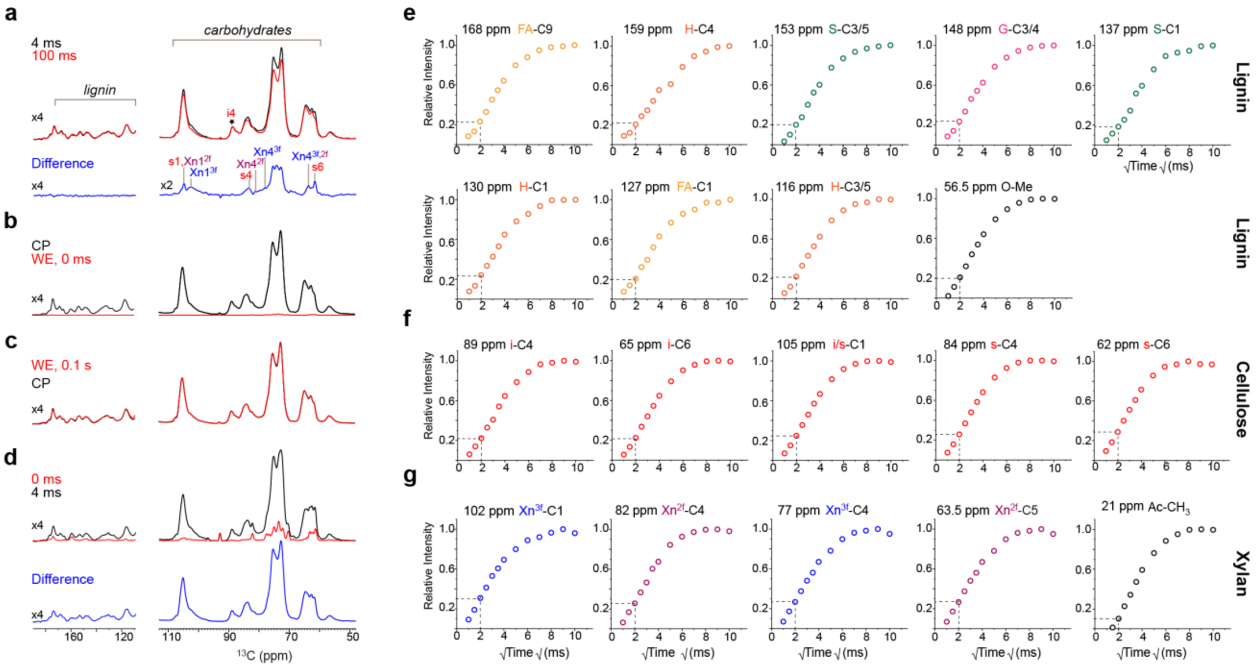

Supplementary Figure 6. Water-to-polysaccharide/lignin buildup curves of *Zea mays*. a, Water-edited (WE) spectra measured with different mixing times. The 4-ms spectrum detects the hydrated molecules and the 100-ms spectrum report equilibrium intensities. the difference spectrum only shows well-hydrated molecules, thus are lacking the hydrophobic lignin and cellulose. **b**, More than 98% of cell wall signals are removed by a ^{1}H-T$_2$ filter in the water-edited spectrum without ^{1}H-mixing. **c**, The 100 ms ^{1}H-mixing restores the equilibrium intensity in the water-edited ^{13}C spectrum (red) with a spectral pattern identical to the equilibrium intensity of the CP spectrum (black). **d**, Subtraction of 1D water-edited ^{13}C spectra with 0 (red) and 4 ms (black) helps eliminate the original signal that survives through the ^{1}H-T$_2$ filter. The water ^{1}H spin diffusion curves for **e**, lignin, **f**, cellulose and **g**, xylan are shown. Dashlines indicate the intensities of 4-ms spectra. The best-hydrated 3-fold xylan has the fastest spin diffusion from water, with ~30% of the equilibrium intensity detected at 4-ms 1H mixing, followed by the 2-fold xylan and surface cellulose (~25%), and then interior cellulose and lignin (~20%). Lignin and internal cellulose are most hydrophobic. These intensities are clearly weaker than those of pectin (60-80%) and cellulose (30-40%) in primary cell walls.

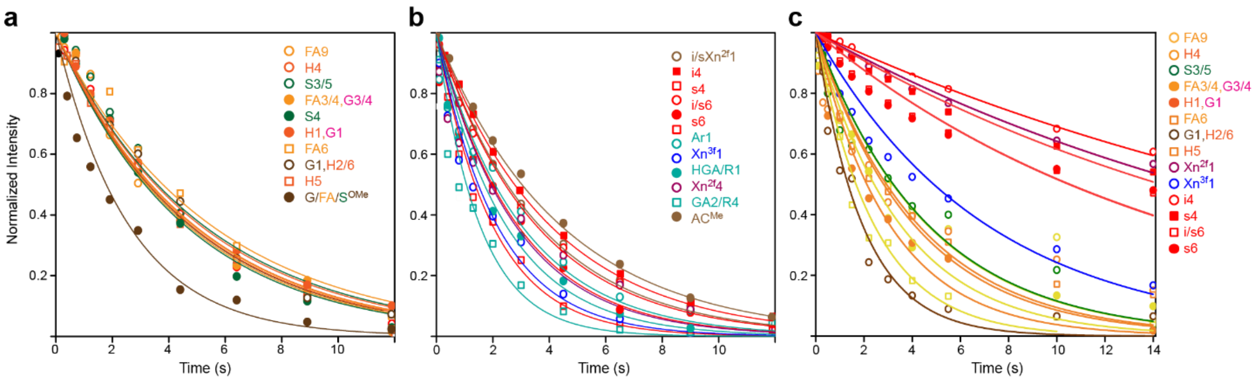

Supplementary Figure 7. Representative ^{13}C-T$_1$ and ^{1}H-T$_{1\rho}$ relaxation curves for *Zea mays*. The ^{13}C-T$_1$ relaxation curves of **a**, lignin and **b**, polysaccharides are shown. **c**, ^{1}H-T$_{1\rho}$ relaxation curves of lignin and polysaccharides. The data are collected on a 400 MHz spectrometer and are fitted using single exponential equations: $I(t) = 1 - e^{-t/T_{1b}}$. The fit parameters are summarized in **Supplementary Table 8-10**. Source data are provided as a Source Data file.

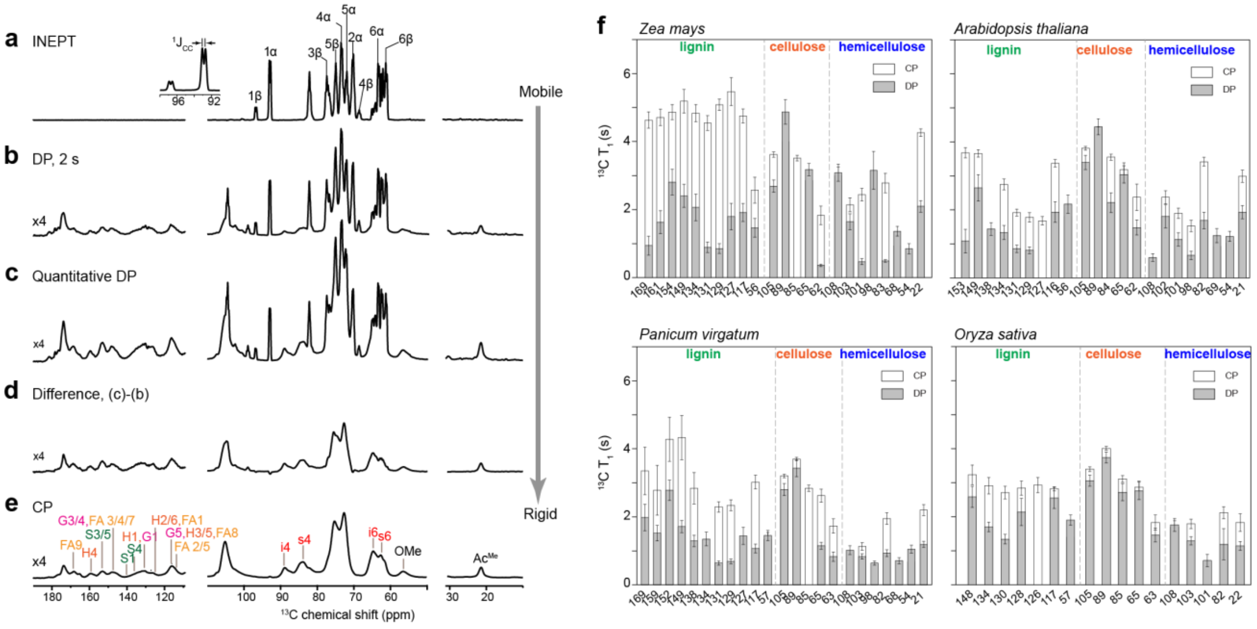

Supplementary Figure 8. The dynamical profile of molecules in intact *Zea mays* stems as resolved using 1D ^{13}C spectra and NMR relaxations. a, ^{13}C INEPT spectrum shows only the signals of highly mobile carbohydrates such as the α- and β-glucose. ^{13}C-^{13}C J-couplings can be resolved as shown. **b**, ^{13}C DP spectrum measured with 2-s recycle delays detects mobile molecules showing major signals from glucose, moderate signals of lignins and starch, and minor signals of polysaccharides. **c**, Quantitative ^{13}C DP measured with 40-s recycle delays. **d**, The difference of ^{13}C DP spectra with 35-s and 2-s recycle delays shows signals of the rigid components. **e**,^{13}C CP spectrum detects the rigid molecules of the cell walls. Substantial signals of lignins are identified in both 2-s DP and CP spectra, thus lignin is dynamically heterogeneous. **f**. ^{13}C-T_1 relaxation times of lignin and polysaccharides in four plants. The open and filled bars represent the ^{13}C-T_1 relaxation times measured using Torchia CP (open) and the standard inversion recovery with quantitative DP (filled). The CP version preferentially detects the rigid molecules and the DP version provides quantitative detection. Error bars are standard deviations of the fitting parameters. Lignins show the highest variation of ^{13}C-T_1 in the CP and quantitative DP detection, indicating the presence of both highly mobile and rigid domains. Source data are provided as a Source Data file.

Supplementary Table 1. Cellulose composition in the primary and secondary cell walls of *Zea mays.* The percentages are calculated using the integrated intensities of C4-C1 and C4-C6 cross peaks. The glucose C6 conformation is an indicator of the hydrogen bonding pattern among glucan chains within the microfibril[1]. Among the seven major cellulose allomorphs, the internal chains a-e adopt trans-gauche (tg) conformation while surface residues g and f primarily adopt the gt conformation, with minor contributions from gg conformers[2]. The increased intensity of type-c cellulose, the dehydrated chains in tg conformation[2,3], indicate conversion of surface chains in gt/gg conformation into tg conformers on the aggregation interface of two cellulose microfibrils.

crystalline and disordered cellulose							
	a	b	c	d	e	f	g
coleoptile	9%	11%	**3%**	8%	2%	31%	36%
stem	9%	19%	**6%**	5%	1%	27%	34%
crystalline cellulose							
	a	b	c	d	e		
coleoptile	27%	32%	**10%**	24%	6%		
stem	22%	47%	**15%**	12%	4%		

Supplementary Table 2. ^{13}C chemical shifts of polysaccharides and lignins in secondary plant cell walls. Superscripts are used to denote different allomorphs. Underline denotes the ^{13}C connectivity with ambiguity. Weak signals or minor species are indicated using "w." Not applicable (/). Unidentified (-).

	**Lignins**	C1	C2	C3	C4	C5	C6	OMe	C7	C8	C9	Plant
H	*p*-hydroxyphenyl	131.0	126.9	116.0	159.4	116.0	126.9	/	/	/	/	*Z. mays*
H		130.2	127.0	116.3	159.5	116.3	127.0	/	/	/	/	*P. virgatum*
G^{a}		130.1	113.8	148.5	148.5	115.0	-	56.1	/	/	/	*Z. mays*
G^{b}	guaiacyl	134.6	-	148.5	148.5	-	-	56.1	/	/	/	*Z. mays*
G^{a}		132.1	114.5	146.2	146.2	114.5	-	56.7	/	/	/	*A. thaliana*
G^{b}		134.8	111.5	147.4	147.4	115.8	120.4	56.7	/	/	/	*A. thaliana*
G		132.7	113.3	148.4	148.4	-	120.5	57.0	/	/	/	*P. virgatum*
G		131.6	-	148.2	148.2	115.5	119.6	56.9	/	/	/	*O. sativa*
S	syringyl	136.5	104.1	153.1	134.3	153.1	104.1	56.1	/	/	/	*Z. mays*
S		136.2	105.5	152.7	134.5	152.7	105.5	56.7	/	/	/	*A. thaliana*
S		137.5	104.9	153.4	133.5	153.4	104.9	57.0	/	/	/	*P. virgatum*
S		135.3	-	153.4	133.3	153.4	-	56.9	/	/	/	*O. sativa*
FA^{a}	ferulate	126.0	114.7	147.8	147.8	114.7	130.4	56.5	147.8	116.0	168.8	*Z. mays*
FA^{b}		126.3	115.0	145.5	145.5	115.0	130.6	56.4	145.5	116.3	168.0	*Z. mays*
FA		128.9	-	148.0	148.0	-	-	57.0	148.0	-	169.4	*P. virgatum*
FA		129.0	115.5	147.9	147.9	115.5	127.4	56.9	-	116.7	169.7	*O. sativa*
	**Polysc.**	C1	C2	C3	C4	C5	C6	AC^{CO}	AC^{Me}			
Xn^{2f}	2-fold xylan	-	-	74.4	82.7	64.9	/	-	-			*Z. mays*
Xn^{2f}	2-fold xylan	105.1	72.5	73.5	82.3	64.6	/	174.0	21.0			
Xn^{2f}	2-fold xylan	105.1	72.5	74.0	81.5	64.0	/	-	-			
Xn^{3f}	3-fold xylan	102.5	73.5	74.5	78.0	62.5	/	174.0	21.0			
	**Cellulose**	C1	C2	C3	C4	C5	C6					
i	type a	105.8	71.5	75.8	89.1	72.5	64.9					*Z. mays*
i	type b	105.1	72.9	74.1	89.1	72.5	65.0					
i	type c	104.2	71.1	-	87.9	-	65.8					
i	type d	105.4	72.8	75.2	87.2	72.5	64.9					
i	type e (w)	105.4	72.1	-	89.9	72.1	65.3					
s	type f	105.2	72.7	75.8	84.3	75.4	62.5					
s	type g	106.1	73.0	73.1	83.5	74.6	61.5					

Supplementary Table 3. Relative intensities of intermolecular cross peaks of *Zea mays.* The intensities are relative ratios of the peak area normalized by the integral of a ^{13}C cross-section. For gated 1-s PDSD peaks higher than 5% are categorized as strong restraints (s, in bold), >2.5% for medium (m) restraints and below 2.5% for weak (w) restraints. All the peaks in the gated 100-ms PDSD spectrum are strong restraints. In total, 74 restraints are identified, including 26 strong restraints, 24 medium ones and 24 weak restraints.

	Atom1 (ω_1)	Atom2 (ω_2)	Gated 100 ms PDSD (%)	Gated 1s PDSD (%)	Type
lignin-cellulose	S/G/FA^{OMe}	i4		0.69	w
	S3/5	i4		2.66	m
	S3/5	i6	**1.91**	3.44	s
	S/G/FA^{OMe}	s4	**0.79**	1.25	s
	S3/5	s6	**1.27**	2.19	s
	FA9	s4		2.21	w
xylan-cellulose	Ac^{Me}	i4		0.79	w
	Ac^{Me}	s4		2.01	w
	Ac^{CO}	i4		0.50	w
	Ac^{CO}	s4		1.90	w
lignin-lignin	S/G/FA^{OMe}	H4		0.63	w
	H1,G1	FA9		4.57	m
	H1,G1	G3/4,FA3/4		**7.84**	s
	H1,G1	S2/6		**5.13**	s
	S4	FA9		**5.37**	s
	S4	H3/5,FA8,G5		**5.42**	s
	S1	G3/4,FA3/4		4.81	m
	G3/4,FA3/4	S3/5		**7.25**	s
	G3/4,FA3/4	S4		**5.57**	s
	S3/5	FA9		2.71	m
	S3/5	H4		1.49	w
	S3/5	G3/4,FA3/4		3.54	m
	S3/5	H1,G1		2.84	m
	S3/5	H2/6		3.31	m
	H4	S3/5		4.40	m
	H4	G3/4,FA3/4		4.20	m
	FA9	S3/5		2.27	w
syringyl-xylan	Ac^{Me}	S3/5		0.83	w
	Ac^{Me}	S4		0.66	w
	S4	Ac^{Me}		3.49	m
	S1	Xn1^{3f}		**8.46**	s
	S1	Xn4^{2f}		3.66	m
	S1	Xn2/3^{3f},Xn3^{2f}		**5.96**	s
	S3/5	Ac^{CO}		1.60	w
	S3/5	Xn1^{3f}		**5.77**	s
	S3/5	Xn4^{2f}	**2.88**	**5.49**	s
	S3/5	Xn4^{3f}		3.09	m
	S3/5	Xn2/3^{3f},Xn3^{2f}	**4.40**	**7.57**	s
	S3/5	Xn5^{2f,3f}	**2.58**	**5.57**	s
	S3/5	Ac^{Me}		3.25	m
	Ac^{CO}	S3/5		0.47	w
p-hydroxyphenyl-xylan	H4	Xn4^{2f}		4.18	m
	H4	Xn4^{3f}		1.99	w
	H4	Xn2/3^{3f},Xn3^{2f}	**2.47**	**17.40**	s
	H4	Xn5^{2f,3f}		**7.36**	s
	H4	Ac^{Me}		**5.33**	s

	Ac^{CO}	H2/6		0.46	w
ferulate-xylan	Ac^{Me}	FA9		1.19	w
	FA9	Ac^{CO}		2.65	m
	FA9	$Xn4^{2f}$		3.22	m
	FA9	$Xn2/3^{3f}$,$Xn3^{2f}$	**5.93**	**27.46**	s
	FA9	$Xn5^{2f,3f}$		**12.04**	s
	FA9	Ac^{Me}	**4.65**	**5.00**	s
OMe-xylan	Ac^{Me}	$S/G/FA^{OMe}$		2.09	w
	$S/G/FA^{OMe}$	Ac^{CO}		1.28	w
	$S/G/FA^{OMe}$	$Xn4^{2f}$	**1.48**	2.76	s
	$S/G/FA^{OMe}$	$Xn4^{3f}$	**0.94**	2.33	s
	$S/G/FA^{OMe}$	$Xn5^{2f,3f}$	**1.99**	**10.26**	s
	$S/G/FA^{OMe}$	Ac^{Me}		3.00	m
	Ac^{CO}	$S/G/FA^{OMe}$		3.07	m
G/FA (ring)-xylan	Ac^{Me}	G3/4,FA3/4		0.48	w
	G3/4,FA3/4	$Xn4^{2f}$		2.63	m
	G3/4,FA3/4	$Xn4^{3f}$		2.54	m
	G3/4,FA3/4	$Xn5^{2f,3f}$		4.23	m
	G3/4,FA3/4	Ac^{Me}		**5.18**	s
lignin (mixed)-xylan	Ac^{Me}	H2/6,FA1,G5		1.14	w
	Ac^{Me}	H3/5,FA8,G5		2.26	w
	H2/6,FA1	$Xn2/3^{3f}$,$Xn3^{2f}$		**5.16**	s
	H2/6,FA1	$Xn5^{2f,3f}$		3.91	m
	H2/6,FA1	Ac^{Me}		2.33	w
	Ac^{CO}	H3/5,FA8,G5		1.58	w
	H1,G1	$Xn5^{2f,3f}$		2.68	m
	H1,G1	Ac^{Me}		3.13	m
	Ac^{CO}	H1,G1		0.46	w

Supplementary Table 4. Relative intensities of intermolecular cross peaks of *Arabidopsis thaliana.* The intensities are relative ratios of the peak area normalized by the integral of a ^{13}C cross-section. For gated 1-s PDSD, a peak higher than 5% is categorized as a strong restraint (s, in bold), >2.5% for medium (m) restraints and below 2.5% for weak (w) restraints. All the peaks in the gated 100-ms PDSD spectrum are strong restraints. In total, 62 restraints are identified, including 23 strong restraints, 16 medium ones and 23 weak restraints.

	Atom1 (ω_1)	Atom2 (ω_2)	100 ms Gated PDSD (%)	1s Gated PDSD (%)	Type
G/S-cellulose	S/G^{OMe}	i4	**1.70**	1.01	s
	S/G^{OMe}	s4	**1.48**	2.19	s
	S4	i4		2.10	w
	S4	s4		4.39	m
	S1	i6		2.11	w
	S3/5	i4		1.05	w
	S3/5	s4		4.33	m
	S3/5	i6		2.53	m
xylan-cellulose	Ac^{Me}	i4		1.06	w
	Ac^{Me}	s4	**0.62**	4.73	s
	Ac^{CO}	i4		1.65	w
	Ac^{CO}	s4		1.79	w
lignin-lignin	S4	G3/4	**5.31**	**5.94**	s
	G3/4	S3/5	**1.04**	1.47	s
	G3/4	S4	**1.09**	1.68	s
	G3/4	S2/6,Xn1^{3f}		3.72	m
	S3/5	G3/4	**4.31**	2.59	s
	S3/5	G6		3.02	m
	S3/5	G2		0.78	w
Guaiacyl (ring)-xylan	Ac^{Me}	G1		0.50	w
	G1	Ac^{CO}		**5.37**	s
	G1	Xn5^{2f,3f}		**5.34**	s
	G1	Ac^{Me}		**5.83**	s
	Ac^{CO}	G3/5		0.63	w
	Ac^{CO}	G1	**0.45**	1.53	s
	Ac^{CO}	G5		0.34	w
	Ac^{CO}	G2		0.46	w
	G3/4	Ac^{CO}		4.46	m
	G3/4	Xn4^{2f}		4.11	m
	G3/4	Xn4^{3f}		1.73	w
	G3/4	Xn5^{2f,3f}	**0.89**	**8.42**	s
	G3/4	Xn2/3^{2f,3f},Xn3^{3f}		**19.52**	s
	G3/4	Ac^{Me}		3.43	m
	Ac^{Me}	G3/4		1.00	w
syringyl-xylan	Ac^{Me}	S1		0.50	w
	Ac^{Me}	S4		0.74	w
	S4	Ac^{CO}		4.13	m
	S4	Xn1^{3f}		1.93	w
	S4	Xn2/3^{2f,3f},Xn3^{3f}		**11.68**	s
	S4	Xn4^{3f}		**6.70**	s
	S4	Xn5^{2f,3f}		**6.81**	s
	S4	Ac^{Me}		3.10	m
	S1	Ac^{CO}		**6.77**	s
	S1	Xn4^{2f}		4.29	m
	S1	Xn5^{2f,3f}		4.14	m

	S1	Ac^{Me}		3.22	m
	S3/5	Ac^{CO}		2.07	w
	S3/5	$Xn4^{2f}$		2.51	m
	S3/5	$Xn4^{3f}$		1.97	w
	S3/5	$Xn5^{2f,3f}$	**2.37**	**5.75**	s
	S3/5	$Xn2/3^{2f,3f}$,$Xn3^{3f}$		**20.69**	s
	S3/5	Ac^{Me}		**5.26**	s
	Ac^{CO}	S3/5		0.57	w
	Ac^{CO}	S1		0.59	w
	Ac^{CO}	S4		0.86	w
	S/G^{OMe}	Ac^{CO}		4.55	m
	S/G^{OMe}	$Xn1^{3f}$		2.23	w
	S/G^{OMe}	$Xn4^{2f}$		2.05	w
OMe-xylan	S/G^{OMe}	$Xn4^{3f}$	**2.02**	1.90	s
	S/G^{OMe}	$Xn2/3^{2f,3f}$,$Xn3^{3f}$	**3.89**	**18.07**	s
	S/G^{OMe}	$Xn5^{2f,3f}$	**2.04**	**7.06**	s
	S/G^{OMe}	Ac^{Me}		2.62	m

Supplementary Table 5. Relative Intensities of intermolecular cross peaks of *Panicum virgatum*. The intensities are relative ratios of the peak area normalized by the integral of a ^{13}C cross-section. For gated 1-s PDSD, a peak higher than 5% is categorized as a strong restraint (s, in bold) and >2.5% for medium (m) restraints and below 2.5% for weak (w) restraints. All the peaks in the gated 100-ms PDSD spectrum are strong restraints. In total, 59 restraints are identified, including 19 strong restraints, 13 medium ones and 25 weak restraints.

	Atom1 (ω_1)	Atom2 (ω_2)	Gated 100 ms PDSD (%)	Gated 1s PDSD (%)	Type
lignin-cellulose	S/G/FA^{OMe}	i4		1.76	w
	H1,G1	i4		1.46	w
	H4	i3		**11.06**	s
	H4	i6		2.60	w
	G3/4,FA3/4	i4		2.17	w
	S/G/FA^{OMe}	s4		2.85	w
	S3/5	s4		3.28	m
xylan-cellulose	Ac^{Me}	i4		0.94	w
	Ac^{Me}	s4		2.60	m
	Ac^{CO}	i4		0.70	w
	Ac^{CO}	s4		4.00	m
lignin-lignin	S1	G2		3.45	m
	S1/4	H1,G1		**6.34**	s
	S3/5	G3/4,FA3/4		1.98	w
	S3/5	G1		2.19	w
	S3/5	H2/6		2.25	w
	S3/5	H3/5		3.80	m
	S4	FA3/4		3.45	m
	G3/4,FA3/4	H3/5		**5.97**	s
	FA6	S1	**5.46**	2.42	s
	FA6	H3/5		1.16	w
	FA6	G2		1.22	w
	FA9	H3/5		1.53	w
guaiacyl-xylan	Ac^{Me}	G6		0.46	w
syringyl-xylan	Ac^{Me}	S3/5		0.73	w
	S1	Ac^{Me}		**5.46**	s
	S3/5	Xn1^{2f}		**7.55**	s
	S3/5	AC^{CO}		2.47	w
	S3/5	Xn5^{2f,3f}		**11.35**	s
	S4	Xn1^{2f}		**7.39**	s
	S4	Xn4^{2f}		**7.56**	s
	S1	Xn4^{2f}		2.55	m
	S1	Xn1^{2f}		2.95	m
	AC^{CO}	S4		0.19	w
p-hydroxyphenyl-xylan	AC^{Me}	H3/5		0.93	w
	AC^{CO}	H4		0.39	w
	H4	Xn2/3^{3f},Xn2^{2f}		**11.06**	s
	H4	Xn5^{3f}		1.89	w
ferulate-xylan	Ac^{Me}	FA6		1.57	w
	Ac^{CO}	FA6		0.24	w
	FA6	AC^{CO}		3.71	m
	FA6	Xn4^{3f}		**7.05**	s
	FA9	Xn5^{2f,3f}		**6.48**	s
	FA6	Ac^{Me}	**6.14**	4.21	s
OMe-xylan	S/G/FA^{OMe}	Ac^{CO}		3.70	m

	S/G/FA^{OMe}	H3/5		2.66	m
	S/G/FA^{OMe}	Xn3^{2f,3f}		**12.35**	s
	S/G/FA^{OMe}	Xn5^{2f,3f}		**6.23**	s
	S/G/FA^{OMe}	AC^{Me}	**0.30**	2.59	s
	Ac^{Me}	G3/4,FA3/4		0.63	w
	G3/4,FA3/4	Xn1^{2f}	**3.23**	**8.96**	s
	G3/4,FA3/4	Xn3^{2f},^{3f}		**11.83**	s
G/FA (ring)-xylan	G3/4,FA3/4	Xn5^{2f}		**8.03**	s
	G3/4,FA3/4	Ac^{Me}		4.91	m
	G3/4,FA3/4	Ac^{CO}		2.66	m
	G3/4,FA3/4	Xn4^{2f}		2.17	w
	H1,G1	Xn5^{2f}/i6		4.04	m
lignin (mixed)-xylan	H4	S2/6,Xn1^{2f},i1		4.72	m
	AC^{CO}	H1,G1		0.72	w

Supplementary Table 6. Relative Intensities of intermolecular cross peaks of *Oryza sativa.* The intensities are relative ratios of the peak area normalized by the integral of a ^{13}C cross-section. For gated 1-s PDSD, the peaks higher than 5% are categorized as strong restraints (s, in bold) and 2.5% for medium (m) restraints and below 2.5% for weak (w) restraints. All the peaks in the gated 100-ms PDSD spectrum are strong restraints. In total, 39 restraints are identified, including 19 strong restraints, 13 medium ones and 7 weak restraints.

	Atom1 (ω_1)	Atom2 (ω_2)	Gated 100 ms PDSD (%)	Gated 1s PDSD (%)	Type
lignin-cellulose	S/G/FA^{OMe}	s4	**0.91**	3.32	s
	G3/4,FA3/4	s4		3.23	m
	S1	i3,s3		**17.2**	s
	S3/5	s4		4.63	m
xylan-cellulose	Ac^{Me}	s4	**0.95**	**3.68**	s
lignin-lignin	FA6	G6		**6.47**	s
	S1	FA9		2.80	m
	S3/5	G3/4,FA3/4	**1.78**	**11.67**	s
	S3/5	FA1		**8.04**	s
	S3/5	G5,FA2/5		**6.48**	s
guaiacyl-xylan	G1	Ac^{CO}		3.16	m
	G1	Xn3^{2f},Xn2/3^{3f}		**8.02**	s
	G1	Ac^{Me}		1.50	w
syringyl-xylan	S3/5	Ac^{CO}		0.63	w
	S3/5	Xn5^{2f,3f}		**8.60**	s
	S3/5	Xn3^{2f},Xn2/3^{3f}		**21.71**	s
	S1	Xn5^{2f,3f}		**4.95**	m
	Ac^{CO}	S4		2.54	m
	Ac^{Me}	S1		2.51	m
ferulate-xylan	FA9	Xn^{2f}4		2.73	m
	FA6	Ac^{CO}		**5.57**	s
	FA6	Xn3^{2f},Xn2/3^{3f}		**13.37**	s
	FA6	Xn^{2f,3f}5		**6.05**	s
	FA6	Ac^{Me}		3.24	m
	Ac^{CO}	FA6		1.14	w
OMe-xylan	S/G/FA^{OMe}	Ac^{CO}	**2.04**	**3.40**	s
	S/G/FA^{OMe}	Xn3^{2f},Xn2/3^{3f}	**0.43**	**17.51**	s
	S/G/FA^{OMe}	AC^{Me}		1.10	w
	Ac^{CO}	S/G/FA^{OMe}	**3.00**	**4.47**	s
	Ac^{Me}	S/G/FA^{OMe}		3.01	m
G/FA (ring)-xylan	G3/4,FA3/4	Ac^{CO}		2.13	w
	G3/4,FA3/4	Xn5^{2f,3f}		**7.55**	s
	G3/4,FA3/4	Xn3^{2f},Xn2/3^{3f}		**16.96**	s
	G3/4,FA3/4	AC^{Me}		2.50	m
	G3/4,FA3/4	Xn1^{2f}		**6.86**	s
	Ac^{CO}	G3/4,FA3/4		2.40	w
	Ac^{CO}	G5,FA2/5		3.08	m
	Ac^{Me}	G3/4,FA3/4		1.22	w
	Ac^{Me}	FA2/5,G5		3.39	m

Supplementary Table 7. Water-edited intensities of polysaccharide cross peaks from 2D ^{13}C-^{13}C correlations spectra. The relative intensities are obtained by comparing the water-edited and control spectra. Error bars are standard deviations propagated from NMR signal-to-noise ratios.

Type	cross peaks	Intensities	Type	cross peaks	Intensities
Interior cellulose	i4-1[a,b]	0.46±0.08	Surface cellulose	s4-1[f,g]	0.43±0.02
	i4-3[a,b]	0.54±0.04		s4-2/5[f]	0.46±0.04
	i4-2/5[a,b]	0.38±0.04		s4-s5g	0.54±0.04
	i4-6[a,b]	0.38±0.08		s4-2[f],4-2/3[g]	0.46±0.04
	i6-1[a,b,c,d]	0.35±0.04		s4-6[f]	0.41±0.02
	i6-4[a,b]	0.35±0.04		s4-6[g]	0.5±0.1
	i6-4[c,d]	0.36±0.01		s6-1[f,g]	0.54±0.08
	i6-3[a,b,d]	0.42±0.04		s6-4[f,g]	0.50±0.01
	i6-3[c]	0.38±0.04		s6-2/5[f]	0.57±0.01
	i6-2/5[a,b,d],i6-2[c]	0.35±0.08		s6-2[f],s6-2/3[g]	0.58±0.08
Xn^{2f}	Xn^{2f}4-1	0.42±0.04	$Xn^{2f,3f}$ Ac^{Me}	$Xn^{3f}Ac^{Me}$-2, $Xn^{2f}Ac^{Me}$-2/3	0.54±0.08
	Xn^{2f}4-3’	0.54±0.04		$Xn^{2f,3f}Ac^{Me}$-5	0.9±0.2
	Xn^{2f}4-3	0.58±0.04	Lignin	FA8-9	0.3±0.2
	Xn^{2f}4-2	0.60±0.03		FA1-3/4	0.2±0.1
	Xn^{2f}4-5	0.43±0.02		FA3/4/7-9	0.1±0.1
	Xn^{2f}3-1	0.5±0.2		FA^{OMe}-9	0.1±0.1
	Xn^{2f}3-4	0.7±0.1		H1-4	0.4±0.2
	Xn^{2f}3-5	0.5±0.2		H5-4	0.2±0.1
	Xn^{2f}5-1	0.4±0.2		H2-4	0.2±0.1
	Xn^{2f}5-4	0.46±0.08		H3-2	0.3±0.1
$Xn^{2f}Ac^{Me}$	$Xn^{2f}Ac^{Me}$-1	0.30±0.02		S4-3	0.1±0.1
	$Xn^{2f}Ac^{Me}$-4	0.34±0.02		S2/6-3/5	0.2±0.1
Xn^{3f}	Xn^{3f}2/3-1	0.8±0.2		S^{OMe}-2/6	0.3±0.2
	**Xn^{3f}2/3-4**	**1.0±0.2**		S^{OMe}-5	0.1±0.1
	Xn^{3f}2/3-5	0.5±0.1	Lignin (mixed)	FA5-3/4,G5-3/4	0.2±0.1
	Xn^{3f}5-1	0.7±0.2		G1-3/4,FA6-3/4	0.1±0.1
	Xn^{3f}5-4	0.7±0.1		FA8-1,H5-2	0.3±0.1
$Xn^{3f}Ac^{Me}$	$Xn^{3f}Ac^{Me}$-1	0.31±0.08		FA^{OMe}-3/4,G^{OMe}-3/4	0.1±0.1
	$Xn^{3f}Ac^{Me}$-5	0.46±0.04		H^{OMe}-5,G^{OMe}-5,FA^{OMe}-5	0.1±0.1
	$Xn^{3f}Ac^{Me}$-3	0.5±0.2		G^{OMe}-1,S^{OMe}-4	0.1±0.1
Xn^{2f}/Xn^{3f}	$Xn^{2f,3f}$5-3	0.50±0.08			
	Xn^{2f}5-2/3,Xn^{3f}5-2	0.42±0.04			

Supplementary Table 8. ^{13}C-T_1 relaxation times of polysaccharides in the hydrated secondary cell walls of four plants. The experiments are measured using Torchia CP or the standard inversion recovery with quantitative DP. The data are fit using single exponential equation: $I(t) = 1 - e^{-t/T_{1b}}$. Error bars are standard deviations of the fitting parameters. Ar stands for the arabinose, GA stands for galacturonic acid, GlcA stands for glucuronic acid, and R stands for rhamnose, the other names are consistent with that from the main figures.

	Zea mays				*Arabidopsis thaliana*				*Panicum virgatum*				*Oryza sativa*			
	Atom (ppm)	T1, CP (s)	Atom (ppm)	T1, DP (s)	Atom (ppm)	T1, CP (s)	Atom (ppm)	T1, DP (s)	Atom (ppm)	T1, CP (s)	Atom (ppm)	T1, DP (s)	Atom (ppm)	T1, CP (s)	Atom (ppm)	T1, DP (s)
FA9	168	4.6±0.2	168	0.9±0.3	-	-	-	-	169	3.4±0.7	169	2.0±0.4	-	-	-	-
H4	160	4.7±0.2	159	1.6±0.3	-	-	-	-	159	2.8±0.7	159	1.5±0.2	-	-	-	-
S3/5	154	4.9±0.2	154	2.8±0.4	153	3.7±0.2	153	1.1±0.4	152	4.3±0.7	153	2.8±0.3	-	-	-	-
G3/4,FA3/4	149	5.2±0.3	149	2.4±0.3	149	3.7±0.1	149	2.7±0.4	149	4.3±0.7	149	1.7±0.2	148	3.2±0.3	148	2.6±0.3
S1	138	-	-	-	-	-	136	1.4±0.2	138	2.8±0.5	137	1.3±0.2	-	-	-	-
S4	134	4.8±0.3	134	2.1±0.4	134	2.8±0.2	133	1.3±0.2	-	-	135	1.4±0.2	134	2.9±0.2	134	1.7±0.1
H1,G1	131	4.5±0.2	131	0.9±0.2	131	1.9±0.1	130	0.9±0.1	130	2.3±0.2	130	0.64±0.06	130	2.7±0.2	130	1.3±0.1
FA6	129	5.1±0.2	129	0.9±0.1	129	1.8±0.1	129	0.8±0.1	129	2.3±0.2	129	0.69±0.07	128	2.8±0.2	128	2.1±0.4
FA1,H2/6	127	5.5±0.4	126	1.8±0.4	127	1.7±0.1	-	-	-	-	126	1.4±0.3	126	2.9±0.2	-	-
H5	117	4.8±0.2	117	1.9±0.3	116	3.4±0.1	117	1.9±0.3	117	3.0±0.2	116	1.1±0.1	117	2.82±0.06	117	2.6±0.3
FA/G/S-O^{Me}	56	2.6±0.4	56	1.5±0.3	56	1.8±0.2	57	2.2±0.3	57	1.3±0.2	57	1.5±0.2	57	1.4±0.2	57	1.9±0.2
i/s/Xn^{2f}1	105	3.62±0.08	105	2.7±0.2	105	3.82±0.06	105	3.4±0.2	106	3.20±0.05	105	2.8±0.2	105	3.40±0.07	105	3.1±0.2
i4	89	4.00±0.07	89	3.9±0.4	89	4.41±0.04	89	4.5±0.2	89	3.70±0.05	89	3.4±0.3	89	4.01±0.06	89	3.7±0.2
s4	85	3.51±0.08	-	-	84	3.55±0.09	83	2.2±0.3	85	2.84±0.09	-	-	85	3.1±0.1	85	2.7±0.3
i/s6	65	2.9±0.3	65	2.6±0.2	65	3.2±0.2	65	3.0±0.3	65	2.2±0.2	64	1.2±0.1	65	2.9±0.2	65	2.8±0.3
s6	62	1.8±0.3	61	0.36±0.04	62	2.4±0.4	62	1.5±0.2	63	1.7±0.2	62	0.8±0.1	63	1.8±0.2	63	1.5±0.2
Ar1	108	3.0±0.3	108	2.6±0.3	-	-	108	0.6±0.1	-	-	109	1.0±0.1	108	1.7±0.2	108	1.8±0.2
Xn^{3f}1	103	2.1±0.2	103	1.6±0.2	102	2.4±0.2	102	1.8±0.3	103	1.1±0.1	103	0.83±0.08	103	1.8±0.1	103	1.3±0.1
GA/R1	101	2.4±0.2	101	0.47±0.09	101	1.9±0.2	101	1.1±0.2	-	-	-	-	-	-	100	0.7±0.2
GlcA1	98	-	99	3.2±0.6	98	1.5±0.2	98	0.7±0.1	-	-	97	0.64±0.06	-	-	-	-
Xn^{2f}4	82	2.8±0.3	82	0.49±0.04	82	3.4±0.1	82	1.7±0.3	82	2.0±0.2	81	0.9±0.1	82	2.1±0.2	82	1.2±0.5
GA2,R4	68	0.9±0.2	68	1.4±0.1	69	0.9±0.1	69	1.2±0.2	-	-	68	0.71±0.09	-	-	-	-
--	54	-	54	0.9±0.2	54	1.02±0.08	54	1.2±0.2	54	0.7±0.1	54	1.1±0.1	-	-	-	-
$Xn^{2f/3f}AC^{Me}$	22	4.3±0.1	22	2.1±0.2	21	3.0±0.2	21	1.9±0.2	21	2.2±0.2	21	1.19±0.09	21	1.8±0.3	21	1.1±0.1

Supplementary Table 9. ^{1}H-T$_{1\rho}$ relaxation times of polysaccharides in the hydrated secondary cell walls of four plants. The data are fit using single exponential equation: $I\left(t\right)=e^{-t/T_{1\rho,\mathrm{b}}}$. Error bars are standard deviations of the fitting parameters.

	Zea mays		*Arabidopsis thaliana*		*Panicum virgatum*		*Oryza sativa*	
	Atom (ppm)	T_1 (ms)	Atom (ppm)	T_1 (ms)	Atom (ppm)	T_1 (ms)	Atom (ppm)	T_1(ms)
FA9	169.2	4.6±0.5	168.9	-	169.8	3.7±0.3	168.0	-
H4	160.4	4.2±0.6	160.4	-	160.4	-	159.0	-
S3/5	153.4	4.6±0.4	152.5	13±1	152.8	3.9±0.4	153.0	-
G3/4,FA3/4	147.5	3.5±0.3	148.6	12±1	148.1	4.8±0.3	148.0	5.4±0.3
H1,G1	130.4	3.1±0.2	130.7	4.2±0.3	130.0	3.3±0.2	129.6	4.5±0.2
FA6	128.9	2.4±0.2	128.9	-	128.5	3.0±0.2	128.0	4.7±0.2
FA1,H2/6	127.3	1.9±0.3	127.3	-	127.3	-	126.3	4.5±0.2
H5	116.6	4.1±0.3	116.6	-	116.8	3.6±0.3	116.5	4.7±0.2
FA/S/G-OMe	57.0	-	57.0	8.4±0.8	56.8	5.7±0.5	57.0	9±1
i/s/Xn1^{2f}	105.0	22.6±0.7	105.0	41±2	105.0	27.4±0.7	105.1	22.7±0.7
i4	89.0	26.9±0.6	89.0	44±2	89.0	26±1	88.9	27.1±0.7
s4	84.5	20.7±0.7	84.1	33±2	84.5	20±1	84.5	20.4±0.8
i/s6	64.8	15.2±0.9	65.2	35±3	64.8	18±2	65.0	17±1
s6	61.5	15±1	62.5	30±3	62.7	15±1	62.5	14±1
Ar1	108.0	-	108.0	-	108.0	-	108.2	7.9±0.8
Xn1^{3f}	102.2	7.1±0.7	102.2	-	102.0	4.6±0.4	103.0	5.8±0.6
GA/R1	100.2	-	100.2	-	100.2	-	100.2	1.7±0.2
Xn4^{2f}	82.0	-	82.0	-	81.8	10.3±0.9	82.5	7.9±0.9
GA2,R4	68.3	-	68.3	-	68.3	-	68.3	2.6±0.3
Xn^{2f,3f}-AC^{Me}	21.0	-	21.0	17±1	21.5	7.4±0.6	-	6.2±0.7

Supplementary Table 10. ^{13}C-T_1 relaxation times of polysaccharides in dried and hydrated *Zea mays*. The data are obtained by measuring a series of 2D ^{13}C-^{13}C correlation spectra with various z-filter time. The data are fit using single exponential equations: $I(t)=1-e^{-t/T_{1b}}$. Error bars are standard deviations of the fitting parameters.

Type	Cross peaks	Dry T1 (s)	Hydration T1 (s)
Interior cellulose (i)	i5-1	4.5±0.3	3.5±0.2
	i3-4	4.2±0.3	2.8±0.4
	i2/5-4	4.2±0.2	3.7±0.2
	i4-1	4.7±0.2	3.2±0.4
	i1-4	4.0±0.2	3.2±0.3
	i1-6	4.5±0.2	4.3±0.1
	i6-3	5.0±0.3	3.7±0.2
	i5-2	3.5±0.1	3.9±0.1
	i5-6	5.0±0.3	3.9±0.1
	i4-6	4.5±0.2	3.7±0.4
	i4-2/5	3.6±0.1	3.8±0.2
	i6-4	3.7±0.4	3.5±0.4
	i3-4	4.3±0.4	2.5±0.5
Surface cellulose (s)	s6-3/5	4.3±0.3	3.0±0.3
	s4-1	4.2±0.3	3.4±0.2
	s4-3	3.9±0.2	3.08±0.09
	s4-5	4.2±0.3	3.7±0.2
	s1-6	4.3±0.2	3.9±0.1
	s1-4	4.1±0.3	3.2±0.3
	s4-6	4.2±0.3	3.9±0.2
	s4-6	4.3±0.3	3.1±0.2
	s6-4	4.2±0.4	2.8±0.3
	s3/5-4	4.0±0.3	3.2±0.3
	s2-3/5	3.9±0.3	3.4±0.2
	s2-4	3.6±0.4	3.2±0.3
i/s	i3-1,s3/5-1	4.3±0.3	3.7±0.4
	i1-3,s1-3/5	4.0±0.3	3.6±0.2
Xn	$Xn^{2f,3f}1$-AC^{Me}	2.3±0.6	3.1±0.6
	$Xn^{2f}6$-AC^{Me}	4.1±0.4	3.5±0.6
	$Xn^{2f,3f}3$-AC^{Me}	4.4±0.5	3.4±0.5
	$Xn^{2f}3$-AC^{CO},$Xn^{3f}2/3$-AC^{CO}	4.7±0.3	2.9±0.5
	$Xn^{2f,3f}6$-AC^{CO}	3.0±0.6	2.7±0.5
	$Xn^{2f}5$-4	4.1±0.1	2.7±0.6
	$Xn^{3f}5$-1	4.1±0.3	2.6±0.4
	$Xn^{2f}5$-AC^{Me}	2.9±0.4	2.5±0.3
	$Xn^{2f,3f}3$-AC^{Me}	4.3±0.7	3.2±0.3
	$Xn^{2f,3f}3$-AC^{CO}	4.0±0.4	2.8±0.6
	$Xn^{2f}5$-AC^{CO}	3.8±0.4	3.2±0.4
	$Xn^{2f}4$-5	4.5±0.2	4.9±0.3
Xn/i/s	$Xn^{3f}5$-2,s6-2	4.27±0.08	2.8±0.3
	$Xn^{2f}2$-1,i2/5-1,s2-1	4.0±0.3	3.9±0.2
	$Xn^{2f}1$-3,s1-3	4.1±0.3	4.07±0.03
	$Xn^{3f}2$-5,s2-6	4.5±0.2	4.7±0.5
	$Xn^{2f}2$-5,i2-6	4.5±0.2	4.7±0.2
	$Xn^{3f}3$-5,s3/5-6	4.7±0.2	3.1±0.1
	$Xn^{2f}5$-2,i6-2/5	4.6±0.4	4.2±0.2
Lignin	H2/6-4	8.8±0.9	3.5±0.5
	FA1-3/4	6.1±0.5	4.5±0.3

	S1-3/5	7.3±0.7	5.7±0.5
	S3/5-1	6.3±0.6	3.0±0.1
	FA3/4-1	7.8±0.6	4.8±0.3
	H4-2	8.9±0.9	4.5±0.4
	FA2/5-9	7.7±0.4	5.2±0.4
	H3/5-4	8.9±0.3	4.5±0.3
	G5-3/4,FA5-3/4	7.3±0.3	5.5±0.5
	G3/4-5,FA3/4-5	7.5±0.4	4.8±0.3
	H4-3/5	7.3±0.3	4.9±0.3

Supplementary References

1. Jarvis M. C. Structure of native cellulose microfibrils, the starting point for nanocellulose manufacture. *Philos. Trans. Royal Soc. A* **376**, (2018).

2. Phyo P., Wang T., Yang Y., O'Neill H., Hong M. Direct Determination of Hydroxymethyl Conformations of Plant Cell Wall Cellulose Using ^{1}H Polarization Transfer Solid-State NMR. *Biomacromolecules* **19**, 1485-1497 (2018).

3. Wang T., Yang H., Kubicki J. D., Hong M. Cellulose Structural Polymorphism in Plant Primary Cell Walls Investigated by High-Field 2D Solid-State NMR Spectroscopy and Density Functional Theory Calculations. *Biomacromolecules* **17**, 2210-2222 (2016).
